# Infusion of C20:0 ceramide into ventral hippocampus triggers anhedonia-like behavior in female and male rats

**DOI:** 10.3389/fnbeh.2022.899627

**Published:** 2022-08-24

**Authors:** Lubriel Sambolín-Escobales, Adariana Feliciano-Quiñones, Lizmarie Tirado-Castro, Cristina Suárez, Dariangelly Pacheco-Cruz, Nashaly Irizarry-Méndez, Wilfred Fonseca-Ferrer, Anixa Hernández-López, María Colón-Romero, James T. Porter

**Affiliations:** ^1^Division of Pharmacology, Basic Sciences Department, Ponce Research Institute, Ponce Health Sciences University, Ponce, Puerto Rico; ^2^Biology and Biotechnology Department, Pontifical Catholic University of Puerto Rico, Ponce, Puerto Rico; ^3^Biology Department, University of Puerto Rico, Ponce, Puerto Rico

**Keywords:** ceramides, anhedonia model, microglia, ventral hippocampus (VH), despair swim test

## Abstract

Increased long-chain C20:0 ceramides have been found in the serum of patients with depression. Moreover, ceramides are linked with increased microglia reactivity and inflammatory cytokine production, which are associated with depression. Since ceramides can readily cross the blood brain barrier, peripheral C20:0 ceramides could enter the brain, activate microglia, and induce depressive-like behavior. In this study, we determined whether localized infusion of C20:0 ceramides into the ventral hippocampus (VH) of rats is sufficient to activate microglia and induce depressive-like behaviors. Adult male and female rats received infusions of C20:0 ceramides or vehicle solution every other day for 2 weeks. After the third infusion, C20:0-infused animals showed reduced sucrose preference suggesting anhedonia-like behavior. In contrast, infusions of C20:0 ceramides did not affect immobility in the forced swim test or sucrose grooming suggesting that the behavioral effects of ceramides are task dependent. Furthermore, C20:0-infusions did not increase Iba-1 + microglia or inflammatory markers in the VH suggesting that localized increases in C20:0 ceramides in the VH are sufficient to induce anhedonia-like behavior without microglia activation.

## Introduction

As central molecules in the metabolism of sphingolipids, ceramides contribute to cell membrane structure and signaling to trigger the cellular effects produced by cytokines (Singh et al., 1998; Ogretmen and Hannun, 2004). High concentrations of long-chain ceramides (C16:0, C18:0, C20:0) have been found in patients with major depressive disorder (MDD) (Gracia-Garcia et al., 2011), and total ceramides are increased in animal models with depressive-like behavior (Gulbins et al., 2013; Oliveira et al., 2016). In addition, evidence suggests that microglia could play a role in depression (Sild et al., 2017). Microglia express cytokines and participate in synaptic transmission, synapse formation, and neuroplasticity which are all functions that are impaired in major psychiatric conditions such as MDD (Panatier et al., 2006; Wake et al., 2013; Papouin et al., 2017; Singhal and Baune, 2017). Furthermore, overactivation and increased amounts of microglia have been found in post-mortem brain tissue from patients with disorders such as MDD and anxiety (Brites and Fernandes, 2015; Setiawan et al., 2015).

Little is known about the relationship between long-chain ceramides and microglial activation. The accumulation of ceramides can produce inflammatory or anti-inflammatory responses depending on the acyl chain length and can affect neuronal development and synaptic activity (Jung et al., 2013). Short-chain C8 ceramides inhibit the inflammatory reactions to lipopolysaccharide in microglia and induce the release of neurotrophic factors (Nakajima et al., 2002), suggesting that short-chain ceramides reduce the inflammatory phenotype of microglia (Jung et al., 2013). However, the effect of long-chain ceramides on microglia is unknown, but the effect is likely to be important since long-chain ceramides reduce brain slice viability (de Monte, 2012).

In this study we examined if increased long-chain C20:0 ceramides in the ventral hippocampus (VH) are sufficient to induce the expression of microglia-related genes and depressive-like behaviors. To assess this, we infused long-chain C20:0 ceramides into the VH of adult male and female rats and evaluated anhedonia-like and despair-like behavior. The male and female rats showed anhedonia-like behavior but not despair-like behavior after three C20:0 ceramide infusions. However, C20:0 ceramide infusions did not increase the expression of microglia-related genes in the VH. Our findings suggest that C20:0 ceramides selectively produce anhedonia-like behavior without activating hippocampal microglia.

## Materials and methods

### Animals

All animals used for behavioral experiments and tissue collection for molecular analysis were treated according to the legal and ethics requirements of the Institutional Animal Care and Use Committee (IACUC) from Ponce Health Sciences University (PHSU). Male and female 53 to 60-days-old Sprague Dawley rats were obtained from PHSU/PRI Animal Facilities and maintained in standard laboratory conditions (12 h light/dark cycle, 25°C), with food and water provided *ad libitum*.

### Ventral hippocampus cannula implantation and long-chain C20:0 ceramide infusion

Our study consisted of two different experimental protocols. The aim of the first experiment was to determine if infusions of exogenous C20:0 ceramides into the VH induce short- and long-term anhedonia-like and despair-like behavior. In this experiment, adult female and male rats (post-natal day 53–60) were surgically implanted with bilateral cannulas in the VH (AP −5.6 mm; ML ± 4.5 mm; DV −7.5 mm), which were fixed in place using anchor screws and dental cement. After cannula implantation, rats recovered for 2 weeks, during which they received handling during the last 7 days to habituate them to cannula manipulation and reduce stress during infusions. Fourteen days after the VH cannula implantation, male and female rats received seven infusions of either 100 μM long-chain C20:0 ceramides or a vehicle solution every other day (48 h between each infusion). The C20:0 (Cat No. 10724, Cayman Chemical Company) ceramide solution was prepared by diluting the drug in alcohol to produce a 1 mM C20:0 ceramide stock solution in 100% ethanol. The stock solution was diluted in saline 0.9% to a final concentration of a 100 μM C20:0 ceramide and 10% ethanol prior to infusion. The vehicle solution was composed of 10% ethanol in saline 0.9%. Infusions were given at a rate of 0.5 μL per minute per side over a period of 2 min. Solutions were made fresh the day of each infusion.

The second experiment was to determine if VH C20:0 ceramide infusions affect behavioral despair, sucrose grooming, or microglia activation. The experimental protocol is the same as in the first experiment, but animals were administered four infusions of 100 μM long-chain C20:0 ceramides or a vehicle solution.

### Behavioral tests

#### Sucrose preference test

Rats received a SPT 24 h after every infusion of long-chain ceramides or vehicle solution for a total of seven sessions of the SPT to assess anhedonia-like behaviors. First, rats went through 4 days of acclimation to the sucrose solution. On days 12 and 13, male and female rats were exposed to two bottles of water in their home cage. On day 14, one of the bottles of water was switched to a bottle containing a 1% sucrose solution so that the animals acclimated to the sucrose. On day 15, the bottles were changed, so animals did not develop preference for the side where the bottles are located. The day of the test, rats were water and food deprived for 5 h and then the test was performed. This test consisted of giving the rats access to a water bottle and to another bottle that contained 1% of sucrose solution for 1 h. Sucrose solution consumption was measured for 1 h and expressed as percent of sucrose intake over water intake to assess anhedonia-like behavior.

#### Forced swim test

The FST assessed despair-like behavior in the rats by measuring the time spent immobile, swimming, or struggling. Immobility was considered when rats were practically immobile and only doing slight movements to keep the head above water surface. Swimming was evaluated when rats moved all their paws to stay afloat and struggling was considered when rats moved the anterior and posterior paws, but the movement of the upper paws break the water surface. In this test we put the rats in a cylinder (15.75′′ in height and 11.8′′ in diameter) filled with water to measure immobility, swimming and struggling time. In the first experiment, animals were placed in the water for 15 min to acclimate the animal to the apparatus on the first day and then were placed back into the water for another 5 min the next day. The whole session was recorded using the ANY-maze software and immobility, swimming, and struggling behavior were measured during the 5 min of the FST. In the second experiment, animals were placed in the water for 10 min. To allow us to assess the FST within 24 h of the C20:0 ceramide infusion, we did not include the 15-min acclimation period. The whole session was recorded using the ANY-maze software and immobility, swimming, and struggling behavior were measured during the last 5 min of the FST. After exposure to the FST, rats were placed in a warm cage for complete drying and the cylinder water is changed between subjects.

#### Sucrose grooming test

Sucrose grooming test also was used to assess apathy-like behavior of the rats. This test consisted of adding a 10% sucrose solution onto the rat’s snout and dorsal coat using an atomizer spray. The sucrose solution was sprayed 5 times along the dorsal coat of the animal and one time on the snout. After this, we measured the duration of grooming activity, which consists of animal grooming the face, extremities and dorsal and ventral areas of the body. This activity was analyzed during a 5-min interval from a video recording of the animal’s behavior. After each session, the cage was cleaned with alcohol and wiped dry for the next subject to be tested.

### Immunofluorescence

The second cohort of animals was sacrificied 20–30 min after the sucrose grooming task. After sacrifice the brain was extracted, one hemisphere was fixed in formaldehyde for histology assessment and the second hemisphere was used for qPCR analysis. Iba-1 immunofluorescence staining was performed on paraffin-embedded tissue. After brain extraction, a sagittal cut was done and one hemisphere was fixed in 10% paraformaldehyde. Tissue was embedded in paraffin and coronal sections of VH were cut at 4 μm using a microtome. Slides were washed in xylene followed by hydration of tissue with ethanol (CDA-19). Slides were rinsed in 10% PBS and then heated while incubating with antigen retrieval 0.01 M Citrate-EDTA buffer (pH = 6.2) at 90–95 °C for 40 min. Tissues were washed with deionized water and remained in PBS for 5 min. Non-specific binding was prevented by adding protein block (Cat No. 50062Z, Life Technologies, Frederick, MD, United States) for 15 min. Tissues were incubated with an Anti-Iba-1 rabbit polyclonal primary antibody (Cat no. 019-19741, FUJIFILM Wako Pure Chemical Corporation) overnight in a humidified chamber at 4°C. A negative control was included by adding PBS instead of primary antibody. On the second day, slides were washed twice with PBS and incubated with Goat Anti-Rabbit IgG Secondary Antibody conjugated with Alexa Fluor 555 (Cat No. A21429, Invitrogen by Thermo Fisher Scientific) for 30 min at room temperature. This was followed by two PBS washes and incubation with DAPI for 5 min to label the nuclei. Tissues were washed with PBS twice, and slides were mounted with ProLong Gold antifade (Cat No. P36934, Invitrogen by Thermo Fisher Scientific). Two to three representative images per brain sample were taken using an Olympus System Microscope Model BX60 (Olympus Life Sciences Solution). ImageJ software was used to measure the mean fluorescence intensity (Chompre et al., 2019) and to perform individual microglial cell counting. The area fraction and microglia cell counts are reported as mean ± SEM.

### Quantitative PCR

For this, VH was dissected, and the tissue was homogenized by diluting with 700 μL of TRIzol reagent and centrifuging 3 min using magnetic beads. RNA was isolated using miRNeasy Micro Kit from Qiagen (Cat No. 217084). An RNA concentration and purity was estimated using the Thermo Scientific NanoDrop 2000 to calculate appropriate concentration for cDNA synthesis as specified in the manufacture’s manual. Complementary DNA was synthesized using the iScript cDNA Synthesis Kit from Bio-Rad (Cat No. 1708891). Then, the cDNA samples were diluted by a 1:20 factor and RT-PCR was performed using iQ SYBR Green Supermix from BioRad (Cat No. 1708882) to evaluate mRNA expression of Iba-1 (microglial marker) and inflammatory genes (TNF-a, Iba-1, TLR-4, NLRP3, HMBG-1). The RT-PCR sequences of each primer are shown in the Supplementary Table 1. These genes were normalized using GAPDH as a housekeeping gene.

## Statistics

All data was reported as the mean ± standard error of the mean. Ordinary two-way ANOVA with Tukey’s multiple comparison test was used to analyze the sucrose preference test. Unpaired parametric *t*-test with Welch’s correction was used to compare between treatments, vehicle vs. ceramide infused animals for the behavioral analysis, immunofluorescence staining quantification and mRNA expression analysis on VH. All analyses were performed using GraphPad Prism 9.2.0 for Windows.

## Results

### Localized ventral hippocampus infusions of C20:0 ceramides induced anhedonia-like behavior in male and female rats

**FIGURE 1:**
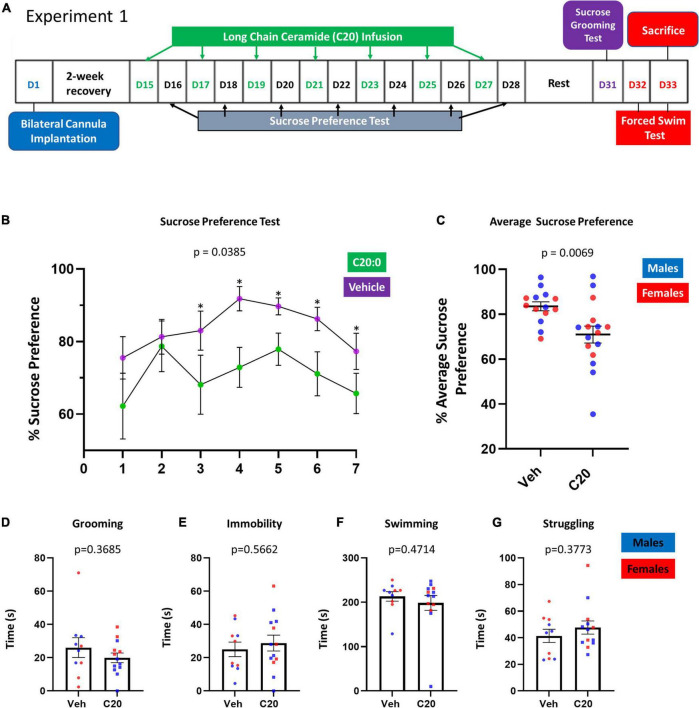
Experimental approach. Long-chain C20:0 ceramide infusions into the VH induced anhedonia-like behavior in male and female rats. **(A)** Timeline of the bilateral cannula implantation, 7–C20:0 ceramide infusions every other day (green font), and intercalated sucrose preference tests 24 h after every infusion (black font). D = day as indicated in the experimental timeline. **(B)** Results of 7 sucrose preference tests given the day after each C20:0 ceramide infusion. **(C)** Average sucrose preference across the 7 sucrose preference tests. **(D)** Sucrose grooming time during 5 min on day 31. **(E–G)** Immobility, swimming, and struggling time measured in the forced swim test to assess despair-like behavior on day 33. *n* = 14 in vehicle group and *n* = 16 in ceramide group. Males in blue symbols and females in red symbols. The symbol * denotes statistical difference, *p* < 0.05.

To determine if the localized infusion of C20:0 ceramides into the VH of rats would cause the development of anhedonia-like behavior, adult male and female rats were implanted with cannulas in the VH and infused with 100 μM C20:0 ceramide or vehicle every other day for 2 weeks. Anhedonia-like behavior was assessed with the SPT 24 h after each VH infusion of C20:0 ceramides as shown in the experimental timeline in Figure 1A. Animals infused with C20:0 ceramides showed less sucrose preference after the third infusion, suggesting an accumulative effect of C20:0 ceramides increased anhedonia-like behavior [Figure 1B, *F*_(6, 6)_ = 4.824, *p* = 0.0385]. Moreover, the animals infused with C20:0 ceramides showed a reduction in the average sucrose preference across the seven C20:0 infusions [Figure 1C, *t*_(22.60)_ = 2.974, *p* = 0.0069] further suggesting that elevated C20:0 ceramides in the VH are sufficient to induce anhedonia-like behavior. Four to 6 days after the last assessment of sucrose preference, rats were exposed to the SGT to assess apathy-like behavior and FST to assess despair-like behavior. The VH infusion of C20:0 ceramides did not affect the grooming activity [Figure 1D, *t*_(13.09)_ = 0.9315, *p* = 0.3685]. Like the SGT, the FST did not show differences in immobility [Figure 1E, *t*_(20.95)_ = 0.5828, *p* = 0.5662], swimming [Figure 1F, *t*_(19.35)_ = 0.7346, *p* = 0.4714], or struggling [Figure 1G, *t*_(20.58)_ = 0.9023, *p* = 0.3773].

### C20:0 ceramide infusions did not affect behavioral despair or sucrose grooming

**FIGURE 2:**
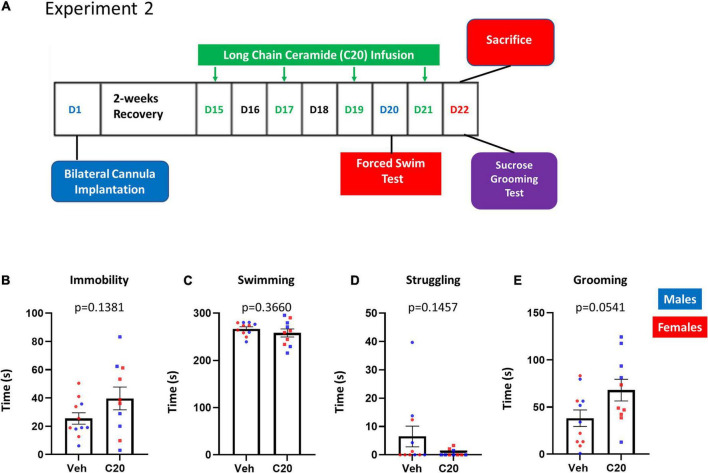
Effects of C20:0 ceramide infusions on despair-like and sucrose grooming behavior. **(A)** Timeline of the bilateral cannula implantation surgery, 4–C20:0 ceramide infusions every 48 h (green font), forced swim test 24 h after the third infusion of C20:0 ceramides (blue font), and sucrose grooming test and sacrifice after the fourth infusion of C20:0 ceramides (red font). D = day as indicated in the experimental timeline. **(B–D)** Immobility, swimming, and struggling time measured during 5 min in the forced swim test to assess despair-like behavior on day 20. **(E)** Sucrose grooming time during 5 min as an assessment of apathy-like behavior on day 22 prior to sacrifice. *N* = 11 in vehicle group and *n* = 10 in ceramide infused groups. Males in blue symbols and females in red symbols.

The lack of effect on the sucrose grooming and behavioral despair in the first experiment could be due to the reversal of the effects of the C20:0 ceramide infusions or to a difference in the sensitivity of the tasks to the ceramides. To distinguish between these possibilities, we exposed a different cohort of animals to the FST and the SGT within 1 day of C20:0 ceramide infusion. Rats received four infusions of C20:0 ceramides every 48 h. Since the sucrose preference in the first experiment was observed the day after the third ceramide infusion, rats were exposed to the FST the day after the third infusion and the SGT after the fourth infusion of ceramides (Figure 2A). The VH infusion of C20:0 ceramides did not affect the immobility [Figure 2B, *t*_(13.27)_ = 1.578, *p* = 0.1381], swimming [Figure 2C, *t*_(13.18)_ = 0.9362, *p* = 0.3660], or struggling time [Figure 2D, *t*_(10.21)_ = 1.575, *p* = 0.1457] in the FST after the third infusion of C20:0 ceramides. In addition, C20:0 ceramides did not affect sucrose grooming after the fourth infusion [Figure 2E, *t*_(17.22)_ = 2.067, *p* = 0.0541]. These data suggest that the behavioral tasks show different sensitivities to the C20:0 ceramide infusions. After the sucrose grooming task, animals were sacrificed, and ventral hippocampal tissue was processed for molecular analysis.

### C20:0 ceramide infusions did not activate ventral hippocampus microglia

**FIGURE 3:**
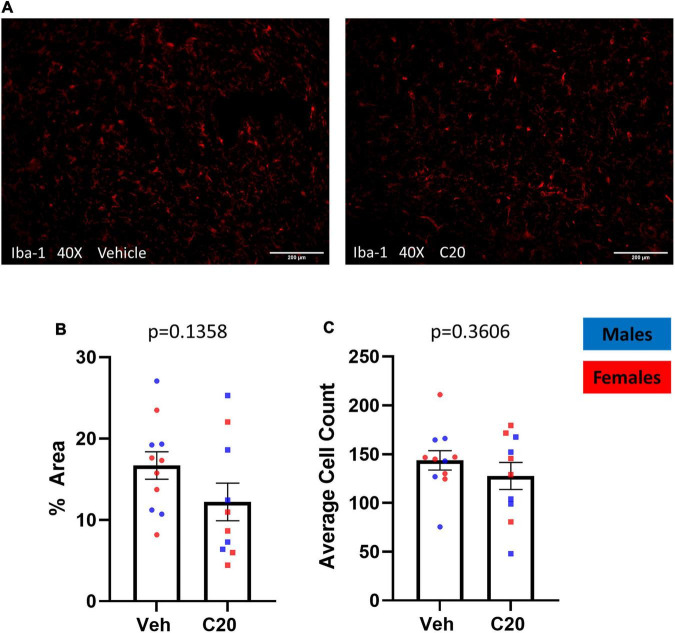
The C20:0 ceramide infusions did not increase microglia in the VH. **(A)** Representative images of VH from vehicle-infused (left column) and ceramide-infused (right column) rats showing microglia labeled with Iba-1. **(B,C)** The expression of the microglial marker Iba-1 in the VH of male and female rats quantified as percent of stained area and number of positive microglia cells. *N* = 11 in vehicle group and *n* = 10 in ceramide infused groups. Males in blue symbols and females in red symbols.

Since ceramides affect cultured microglia (Nakajima et al., 2002; Jung et al., 2013) and microglia are associated with anhedonia-like behavior (Henry et al., 2008), we investigated if the infusion of C20:0 ceramides affected microglia in the VH by labeling the microglial marker Iba-1 in brain tissue collected 20–30 min after the sucrose grooming task (Figure 3A). The infusion of C20:0 ceramides did not affect total Iba-1 protein expression measured as the percent of area labeled [Figure 3B, *t*_(16.90)_ = 1.566 *p* = 0.1358] or the number of Iba-1 + microglia in the VH [Figure 3C, *t*_(16.78)_ = 0.9400, *p* = 0.3606] suggesting that the C20:0 ceramides did not activate VH microglia.

### The infusion of C20:0 ceramides did not induce neuroinflammation in the ventral hippocampus

**FIGURE 4:**
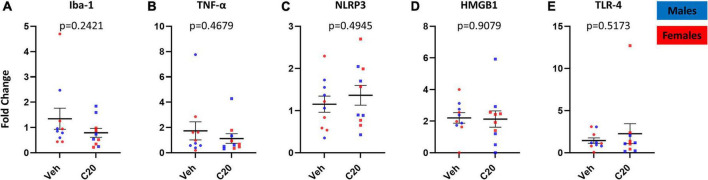
The mRNA expression of inflammatory mediators in the VH 1 day after the last infusion of C20:0 ceramides. **(A–E)** Expression of Iba-1, TNF-α, NLRP3, HMGB1, and TLR-4 mRNA in VH tissue after four infusions of C20:0 ceramides into the VH of male and female rats. *N* = 10 in vehicle group and *n* = 10 in ceramide infused groups. Males in blue symbols and females in red symbols.

In addition to quantifying the VH microglia, we also assessed whether the neuroinflammatory profile in the VH was increased by the infusion of C20:0 ceramides. Consistent with the lack of change in overall Iba-1 protein expression, we found that the ceramide infusion did not change Iba-1 mRNA expression [Figure 4A, *t*_(12.09)_ = 1.230, *p* = 0.2421]. Furthermore, C20:0 infusions did not increase the mRNA expression of the inflammatory cytokine TNF-α [Figure 4B, *t*_(13.71)_ = 0.7466, *p* = 0.4679] or the inflammasome NLRP3 [Figure 4C, *t*_(17.28)_ = 0.6979, *p* = 0.4945]. Moreover, we measured the mRNA expression of the high mobility group box-1 (HMGB-1), which can signal inflammation through activation of the inflammasome and NF-kB signaling cascade (Scaffidi et al., 2002; Yang et al., 2010; Crews et al., 2013; Franklin et al., 2018) and found difference in the ceramide-infused animals [Figure 4D, *t*_(15.43)_ = 0.1176, *p* = 0.9079]. Lastly, we found that the infusion of ceramides did not affect the mRNA expression of the inflammatory modulator TLR-4 [Figure 4E, *t*_(10.30)_ = 0.6705, *p* = 0.5173] suggesting that the infusion of C20:0 ceramides into the VH did not induce neuroinflammation.

## Discussion

Although clinical findings suggest that long-chain C20:0 ceramides may contribute to depression (Gracia-Garcia et al., 2011; Brunkhorst-Kanaan et al., 2019; Hong et al., 2021), it is not clear whether increases in C20:0 ceramides in the brain are sufficient to induce depressive-like behavior in rodents. To address this gap in knowledge, we studied the effects of ventral hippocampal infusion of C20:0 ceramides on different behavioral tasks. We found that infusing C20:0 ceramides into the VH of female and male rats induced anhedonia-like behavior in the sucrose preference task beginning the day after the third infusion. In contrast, animals infused with C20:0 ceramide showed no signs of altered despair-like or apathy-like behavior after three or four infusions, respectively. The behavioral effects of the C20:0 ceramide infusions did not require microglial activation, since the expression of Iba-1 and other microglia-related genes were not increased by the infusions. These findings suggest that anhedonia-like behavior in the sucrose preference test is more sensitive to C20:0 ceramides in the VH and occurs independent of microglial activation.

Research suggests that long-chain ceramides in the hippocampus contribute to depressive-like behaviors in rodents. A previous study found strong evidence that fluoxetine and amitriptyline produce their antidepressant effects by reducing acid sphingomyelinase production of hippocampal ceramides in male mice (Gulbins et al., 2013). Furthermore, direct infusion of long-chain ceramides into the hippocampus are sufficient to induce depressive-like behaviors in rodents. For example, infusing C16:0 ceramides into the dorsal hippocampus caused anhedonia-like (Gulbins et al., 2013) and despair-like (Zoicas et al., 2019) behavior in male mice. We found that infusions of C20:0 ceramides into the VH also induced anhedonia-like behavior as determined by sucrose preference in both male and female rats, while no changes in apathy-like behavior were assessed with the sucrose grooming test. In contrast, infusion of C20:0 ceramides into either the VH in the current study or the dorsal hippocampus (Zoicas et al., 2019) did not affect despair-like behavior in the forced swim task. This suggests that the behavioral effects produced by increases in long-chain ceramides in the hippocampus depend on the ceramide chain length.

Although microglia are associated with anhedonia-like behavior (Henry et al., 2008) and can be affected by short-chain ceramides (Nakajima et al., 2002; Jung et al., 2013), we did not find evidence of activated VH microglia in the animals infused with C20:0 ceramides. Since we did not find evidence of increased expression of inflammatory cytokines in the VH, the microglia did not seem to be producing inflammation but may be primed to produce larger inflammatory responses to subsequent stimulation with factors such as lipopolysaccharide (Jung et al., 2013; Scheiblich et al., 2017). Similarly, the mechanism by which C16:0 ceramides in the dorsal hippocampus induces anhedonia-like behavior is also unknown (Gulbins et al., 2013).

In conclusion, our findings suggest that localized increases in C20:0 ceramides in the VH are sufficient to induce anhedonia-like behavior through a mechanism that does not require microglial activation. Further studies are needed to explore the mechanisms by which different long-chain ceramides produce their behavioral effects.

## Data availability statement

The raw data supporting the conclusions of this article will be made available by the authors, without undue reservation.

## Ethics statement

This animal study was reviewed and approved by the Institutional Animal Care and Use Committee from Ponce Health Sciences University.

## Author contributions

LS-E: conceptualization, methodology, investigation, validation, formal analysis, resources, supervision, writing – original draft, and writing – review and editing. AF-Q: investigation, formal analysis, and writing – original draft. LT-C: investigation, writing – original draft, and writing – review and editing. CS: investigation and writing – review and editing. DP-C and WF-F: writing – original draft. NI-M, AH-L, and MC-R: investigation. JP: conceptualization, methodology, validation, formal analysis, resources, supervision, writing – original draft, and writing – review and editing. All authors contributed to the article and approved the submitted version.
