# Supplementary material for: Infusion of C20:0 ceramide into ventral hippocampus triggers anhedonia-like behavior in female and male rats
Source: Front Behav Neurosci. 2022 Aug 24;16:899627. doi: 10.3389/fnbeh.2022.899627 (PMC9449580; doi:10.3389/fnbeh.2022.899627)
Supplement: Supplementary file 1 [file Table_1.pdf]

Gene	Name	Sequence
**GAPDH**	**Glyceraldehyde-3-Phosphate dehydrogenase**	F: 5’-GAT GCT GGT GCT GAG TAT GT-3’<br>R: 5’-GCG GAG ATG ATG ACC CTT T-3’
**IBA-1**	**Iodized calcium-binding adapter molecule 1**	F: 5’-CCT GAG GAG ATT TCA ACA GAA GC-3’<br>R: 5’-GGA CCG TTC TCA CAC TTC CC-3’
**TNF-α**	**Tumor necrosis factor alpha**	F: 5’-TCT TCT CAT TCC TGC TTG TGG C-3’<br>R: 5’-CAC TTG GTG GTT TGC TAC GAC G-3’
**HMGB-1**	**High-mobility group box 1**	F: 5’-GAG GTG GAA GAC CAT GTC TG-3’<br>R: 5’-AAG AAG AAG GCC GAA GGA GG-3’
**NLRP3**	**NLR family pyrin domain containing 3**	F: 5’-AGA AGC TGG GGT TGG TGA ATT-3’<br>R: 5’-GTT GTC TAA CTC CAG CAT CTG-3’
**TLR-4**	**Toll-like receptor 4**	F: 5’-TCC CTG CAT AGA GGT ACT TC-3’<br>R: 5’-CAC ACC TGG ATA AAT CCA GC-3’

Supplementary Table S1: Primer sequences for the assessment of mRNA levels of microglia cells and inflammatory markers.
